# Effects of *Silybum marianum* L. Seed Extracts on Multi Drug Resistant (MDR) Bacteria

**DOI:** 10.3390/molecules29010064

**Published:** 2023-12-21

**Authors:** Shimaa El-Sapagh, Nanis G. Allam, Mohamed Nour El-Dein El-Sayed, Asmaa Ahmed El-Hefnawy, Grażyna Korbecka-Glinka, Awad Y. Shala

**Affiliations:** 1 Botany and Microbiology Department, Faculty of Science Tanta University, Tanta 31527, Egypt; shimaa.elsapagh@gmail.com (S.E.-S.);; 2 Soil, Water and Environment Research Institute, Agricultural Research Station, Sakha, Kafr El-Sheikh 33717, Egypt; drmohamednour1961@gmail.com; 3 Department of Plant Breeding and Biotechnology, Institute of Soil Science and Plant Cultivation—State Research Institute, Czartoryskich 8, 24-100 Puławy, Poland; 4 Medicinal and Aromatic Plants Research Department, Horticulture Research Institute, Agricultural Research Center (ARC), Giza 12619, Egypt; awad.shala@yahoo.com

**Keywords:** wound infection, multidrug resistant bacteria, antibacterial natural compounds, milk thistle, in-silico, ADMET

## Abstract

Wound infections became a great challenge, especially after the emergence of bacterial resistance to commonly used antibiotics. Medicinal plants can be the source of alternative antibacterial agents effective against multi drug resistant (MDR) bacteria. This research aimed to evaluate the effectiveness of different *Silybum marianum* seed extracts in fighting MDR bacteria that infect wounds. First, thirty purified bacterial cultures obtained from superficial, infected wounds were subjected to antibiotic sensitivity tests. The selected MDR isolates were then used to test the antimicrobial effects of different *S. marianum* seed extracts. The most potent extract was evaluated for its impact on the ultrastructure of the cells of sensitive bacterial isolates using transmission electron microscopy (TEM). The bioactive ingredients of this extract were analyzed by means of gas chromatography–mass spectroscopy (GC–MS). Then, in-silico absorption, distribution, metabolism, excretion, and toxicity (ADMET) properties were predicted for the main components. The results indicated that four out of 30 bacterial isolates were considered MDR bacteria. Primary morphological features of colonies, secondary (automatic) identification using the Biomerieux Vitek 2 System, and 16S rRNA sequencing of the four isolates confirmed that they represent *Staphylococcus aureus*, *Stenotrophomonas maltophilia*, *Klebsiella pneumoniae*, and *Escherichia coli.* Among different extracts of *S. marianum* seeds, ethanol extract showed the strongest inhibitory effect on both Gram-positive and Gram-negative bacteria, with minimum inhibitory concentration (MIC) values between 9.375 and 1.172 mg/mL. However, at concentrations four times higher, this extract was unable to kill bacterial cells, indicating that it had a bacteriostatic effect on the tested MDR strains. TEM revealed denaturation and distorted cell ultrastructure in *S. aureus* and *S. maltophilia* after exposure to ethanol extract. In addition, GC–MS analysis of the ethanol extract identified nine compounds known to have important biological activities, and ADMET analysis showed good drug-likeness for two of these compounds. Consequently, *S. marianum* seeds could be a good source of alternative bacteriostatic agents effective against MDR bacterial strains that cause wound infections.

## 1. Introduction

The skin forms a protective physical barrier, preventing pathogenic invasion [1]. Suppose this skin barrier is cut, torn, or punctured as a result of an accident, trauma, fall, burn, or surgical procedure. In that case, this defense mechanism is disrupted, the skin loses its protective function, and bacteria find a rich environment and perfect conditions (warm, moist, and nutritious) to grow, colonize, proliferate, and cause infection [2]. Wound infections pose a significant risk to human health and can lead to higher mortality rates by hindering the wound-healing process, causing tissue damage, and extending hospital stays and medical treatment costs [3,4,5]. As a result, patients’ overall quality of life is significantly reduced. Although there have been considerable advancements in the technology employed for wound treatment and surgery, wound infections are the main cause of hospital-acquired infections [6] and are responsible for over 80% of the death rate [7]. Wound infection management is extremely challenging, particularly when dealing with multi drug resistant (MDR) bacterial infections [8,9].

Inappropriate use of antibiotics in human and animal healthcare leads to the development of bacterial resistance to available antibiotics [10,11,12]. This is a serious global problem that results in financial and human losses. Bacterial resistance is responsible for the deaths of ~700,000 individuals annually worldwide [13]. Due to the rapid increase in bacterial resistance to antibiotics, the effectiveness of available antibiotics may be lost within 5 years because of the genetic mutations in the resistant bacteria [14]. Additionally, owing to the side effects of some antibiotics and the lack of development and innovation of new antibiotics, humans have started losing their battle against MDR microorganisms. It has become necessary to find new, effective, alternative, and safe antibacterial agents to address this serious problem [15,16,17].

Medicinal plants can be used as alternative antibacterial agents. Although there has been considerable development of conventional medicine, medicinal plants remain an important source for drug discovery owing to their safety and low production cost compared with synthetic medications. Furthermore, medications derived from medicinal plants contain valuable active components with therapeutic benefits, such as flavonoids, alkaloids, terpenoids, carotenoids, polyphenols, saponins, tannins, volatile oils, minerals, and vitamins, as well as potent pharmaceutical properties [18,19]. The World Health Organization reported that approx. 80% of the global population relies on medicinal plants for treating diseases [20]. Furthermore, ~50% of therapeutic agents are derived from natural products [21], and they can serve as raw materials for numerous new medicinal treatments. Using plant extracts as antibacterial agents against wound-infecting bacteria and examining their chemical properties are essential steps in developing new strategies to overcome bacterial resistance. However, it is a challenge to find new sources of raw materials for pharmacological applications [22].

*Silybum marianum* is an important medicinal plant and a promising alternative natural agent with many medicinal uses. The common name of *S. marianum* (L.) is milk thistle; it belongs to the Asteraceae family and is a wild, annual herb found in various areas of the world, including Europe, the Mediterranean, the United States, and South Africa [23]. Milk thistle has many therapeutic properties, so it is used in pharmaceutical applications in some countries. The United States uses milk thistle as a nutritional supplement because it is safe and well-tolerated; it is also recommended as a supplement in Europe [24,25]. *S. marianum* seeds are rich in silymarin, the main active ingredient of milk thistle. Silymarin has numerous pharmaceutical properties and is used for treating liver diseases and promoting liver regeneration owing to its detoxifying activity. Furthermore, it exhibits antiviral activity against hepatitis C and B viruses and anti-inflammatory effects. In addition, it shows considerable anticancer activity against breast, ovarian, and prostate cancers, as well as antimetastatic, antifibrotic, and antiangiogenic effects, and inhibits lipid peroxidation [26,27]. *S. marianum* extracts also demonstrate potent antibacterial properties [28,29]. Therefore, the aim of this research was to determine the antibacterial efficacy of *S. marianum* seeds against wound-infecting MDR isolates using aqueous, ethanol, and acetone extracts.

## 2. Results

### 2.1. Identification and Testing of the Antibiotic Sensitivity of Bacterial Isolates

Pure cultures of 30 bacterial isolates were obtained from superficial, infected wounds. Eighteen (60%) of these isolates were identified as Gram-positive cocci; all of them belonged to the same species—*Staphylococcus aureus* (Table 1). The remaining twelve isolates (40%) were classified as Gram-negative bacilli. The species identified in this group included *Pseudomonas aeruginosa* (6 isolates, 20% of all isolates), *Escherichia coli* (3 isolates, 10%), *Klebsiella pneumoniae* (2 isolates, 6.7%), and *Stenotrophomonas maltophilia* (1 isolate, 3.3%, Table 1).

Disc diffusion tests showed that 30 bacterial isolates differed widely in their sensitivity to the antibiotics tested (Table 1). Four isolates were considered to be highly resistant. From the Gram-negative bacteria, isolates nos. NDL224 (*K. pneumoniae*), NDL225 (*E. coli*), and NDL2210 (*S. maltophilia*) were resistant to 88.9%, 94.4%, and 94.4% of the tested antibiotics, respectively. On the other hand, among Gram-positive bacteria, isolate no. NDL2220 (*S. aureus*) was resistant to 83.3% of the tested antibiotics. The results for these most MDR bacteria were confirmed in automatic antibiotic susceptibility tests carried out by means of VITEK2 Compact_15 for ID&AST (Tables S1 and S2). The isolate no. NDL2220 appeared to be resistant to cefoxitin, benzylpenicillin, and oxacillin, confirming that this isolate was methicillin-resistant *S. aureus* (MRSA), which is considered to be one of the most dangerous bacteria. 

### 2.2. Identification of MDR Bacterial Strains

**Figure 1:**
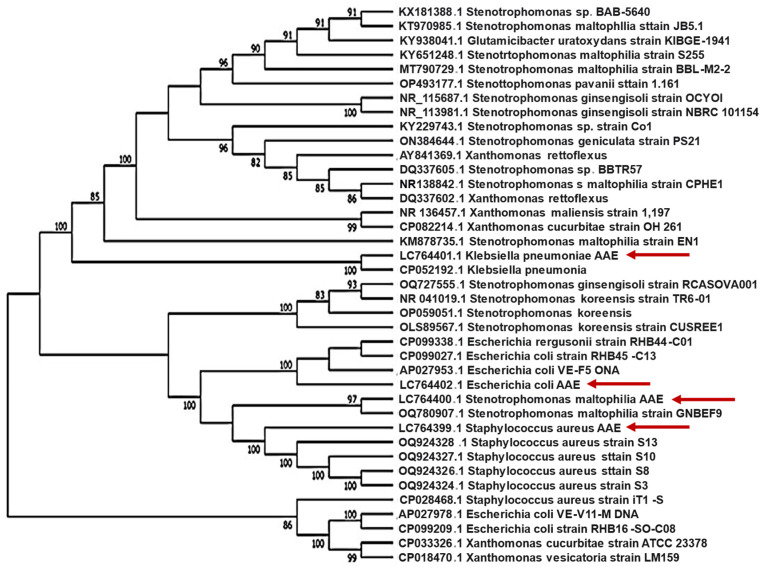
A phylogenetic tree including *Staphylococcus aureus*, *Stenotrophomonas maltophilia*, *Klebsiella pneumoniae*, and *Escherichia coli* based on 16S rRNA sequences. Bootstrap values greater than 70 are shown in the tree. This analysis was conducted using the neighbor-joining method in MEGA 11 software. Red arrows indicate isolates obtained and sequenced in this study.

The highly resistant bacterial isolates (nos. NDL224, NDL225, NDL2210, and NDL2220) were subjected to automated identification using the Biomerieux Vitek 2 System. The results, summarized in Table S3, revealed the following species identification of the bacterial isolates: the isolate no. NDL224 was identified as *Klebsiella pneumoniae* with 98% probability, the isolate no. NDL225 was identified as *Escherichia coli* with 99% probability, the isolate no. NDL2210 was identified as *Stenotrophomonas maltophilia* with 99% probability, and the isolate no. NDL2220 was identified as *Staphylococcus aureus* with 98% probability. Species identification for MDR strains was confirmed via molecular analysis based on 16S rRNA sequencing. BLAST analysis of the sequences obtained for isolates nos. NDL224, NDL225, NDL2210, and NDL2220 showed their high similarity to sequences already available in the NCBI database belonging to *K. pneumoniae*, *E. coli*, *S. maltophilia*, and *S. aureus*, respectively. This result was also confirmed by phylogenetic analysis; the four MDR isolates obtained in this study clustered with NCBI records representing the same species (Figure 1). Therefore, our isolates were named *Klebsiella pneumoniae* AAE, *Escherichia coli* AAE, *Stenotrophomonas maltophilia* AAE, and *Staphylococcus aureus* AAE. Subsequently, their 16S rRNA sequences were deposited at the NCBI under the accession numbers LC764401, LC764402, LC764400, and LC764399, respectively.

### 2.3. Antibacterial Activity of Plant Extracts against MDR Bacteria

*S. marianum* seed ethanol extract showed the strongest antibacterial activity compared to extracts obtained with other solvents. It was effective against all four MDR bacteria, with an average inhibition zone ranging between 21 and 34 mm, depending on the species (Table 2). The acetone extract showed antibacterial activity against MRSA only (with an average inhibition zone of 25 mm), but it did not demonstrate any activity against Gram-negative bacteria. Conversely, the cold water extract displayed antibacterial effects against only Gram-negative bacteria, while the hot water extract did not show any antibacterial activity against any of the tested MDR strains.

### 2.4. MICs of the Most Active Plant Extract against MDR Bacterial Isolates

The ethanol extract showed the highest activity against MDR bacteria. Therefore, it was selected to determine the MIC using the broth microdilution method with TTC. The recorded MIC values ranged between 1.172 and 9.375 mg/mL, depending on the bacterial species (Table 3). The highest MIC values were recorded for *E. coli*, whereas the lowest—for *S. maltophilia*. Broth dilutions up to four times MICs that were plated onto agar plates and incubated still resulted in organism proliferation and did not cause bacterial death. Therefore, it can be concluded that the ethanol extract acts as a bacteriostatic agent.

### 2.5. Effects of S. marianum Seed Ethanol Extract on the Cell Ultrastructure of MRSA and S. maltophilia via TEM

**Figure 2:**
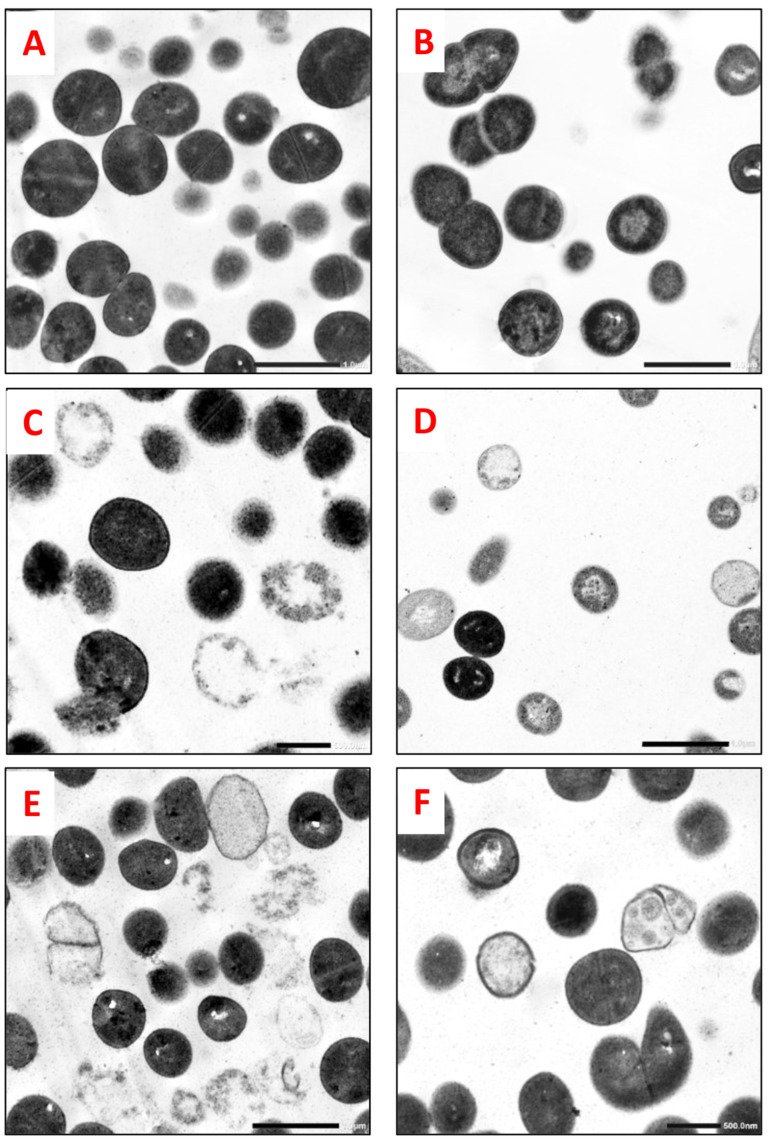
TEM photographs revealed the antibacterial effects of *S. marianum* seed ethanol extract on MRSA (direct magnification: 8000×). (**A**,**B**): Control (normal) cells not treated with the extract displayed a regular cell ultrastructure with a thick cell wall and a regular plasma membrane containing normal organelles. (Note the septa formation during cell division). (**C**–**F**): Cells treated with *S. marianum* seed ethanol extract (2.344 mg/mL) showed abnormal changes leading to denaturation and cell damage. (Note the presence of cell debris in the background of some sections).

**Figure 3:**
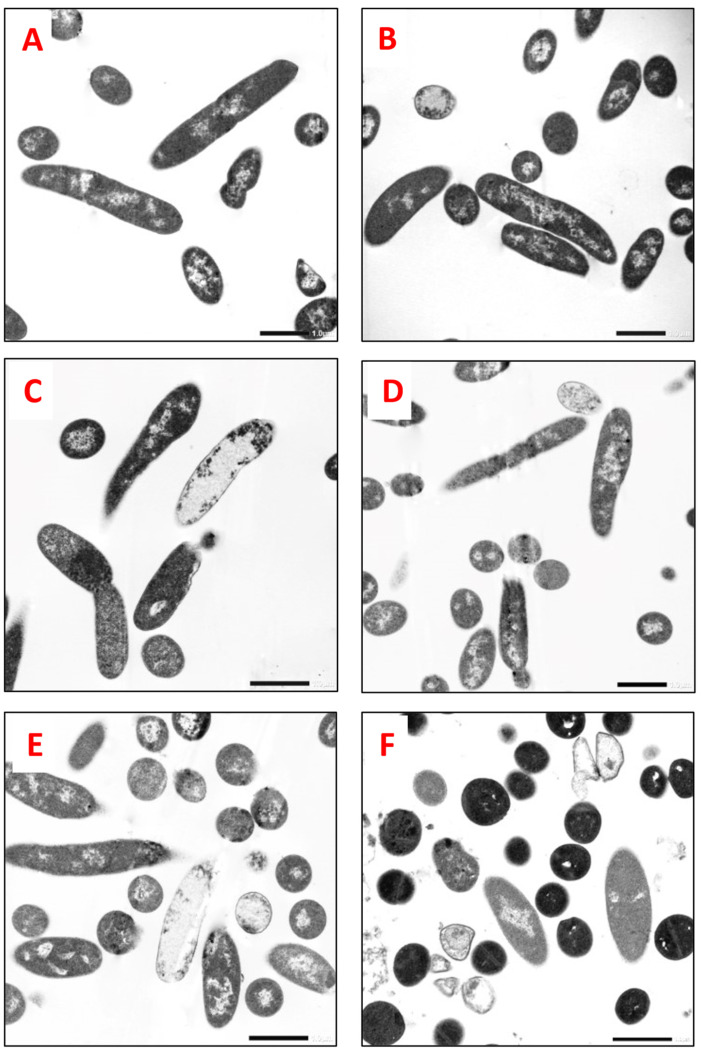
TEM photographs revealed the antibacterial effects of *S. marianum* seed ethanol extract on *S. maltophilia* (longitudinal section and transverse section; direct magnification: 5000×). (**A**,**B**) Control (normal) cells not treated with the extract displayed a normal ultrastructure of Gram-negative bacteria. (**C**–**F**) Cells treated with *S. marianum* seed ethanol extract (1.172 mg/mL) showed denaturation and deformation.

The antibacterial effect of *S. marianum* seed ethanol extract was expressed by bacterial cell rupture, leading to the excessive release of cellular material. Some cells were empty, without organelles, plasmolysis, or vacuolization; irregular shrinkage of the bacterial content to one side of the cell; and cell wall distortion, leading to the complete alteration and collapse of the bacterial cells. Some sections displayed cell debris in the background (Figure 2 and Figure 3) of the treated cells compared with the normal cells (control).

### 2.6. GC–MS

The main bioactive components of the *S. marianum* seed ethanol extract were detected by GC-MS by comparing their peak retention time, height (percentage), and mass spectral fragmentation patterns to those of identified compounds available in the databases of the NIST08, WILEY8, and FAME libraries. The GC–MS chromatograms in Figure S1 and Table S4 displayed the active components alongside their retention time, molecular formula, molecular weight, and peak area percentage. In the ethanol extract of *S. marianum*, analyzed by GC-MS, a total of 29 compounds were identified. Nine of these compounds, each present at 0.5% or more, were considered the main components. The main nine bioactive components can be categorized into four classes: fatty acids, esters, sugars, steroids, and other compounds. The key fatty acids identified are linoleic acid (20%) and linolelaidic acid methyl ester (0.616%). The esters include ethyl linoleate (9.596%), 1-monolinoleoylglycerol trimethylsilyl ether (3.097%), and diisooctyl phthalate (3.276%). The sugar fraction contains d-mannose (4.332%). Other major components are d-mannitol (14.839%), 1-decylsulfonyl-(mandenol) (14.839%), n-methyl-1-adamantaneacetamide (1.876%), and desulphosinigrin (0.830%). All nine components have documented biological activities or medical benefits, including immune-enhancing, antitumor, anticancer, antioxidant, antimicrobial, and anti-inflammatory effects (Table 4). 

### 2.7. ADMET Analysis

Tables S5 and S6 show identified SMILES strings and full data on the prediction of in silico absorption, distribution, metabolism, excretion, and toxicity (ADMET) properties of the main components that were detected by GC-MS. Analysis of the nine bioactive compounds reveals diverse physicochemical properties (Table 5). Molecular weights range from 180.16 to 498.89 g/mol. The heavy atom counts range from 12 to 33, with the lowest count for d-mannose and the highest—for 1-monolinoleoylglycerol trimethylsilyl ether. The Csp3 fraction varies from 0.67 to 1, with 1-monolinoleoylglycerol trimethylsilyl ether being the least saturated. The number of rotatable bonds ranges widely from 1 for d-mannose to 22 for 1-monolinoleoylglycerol trimethylsilyl ether. Hydrogen bonding capacity also differs, with some compounds unable to donate H-bonds while others can donate up to 5. LogS values are consistently negative, reflecting low aqueous solubility, while LogD and LogP values range from 2.499 log mol/L for quite hydrophilic d-mannose to 8.363 log mol/L for very hydrophobic 1-monolinoleoylglycerol trimethylsilyl ether. In summary, this study showed that the compounds have a very acceptable number of donors and acceptors; the first four compounds showed good solubility, while the rest of the compounds showed lower solubility due to their oil nature. 

**Figure 4:**
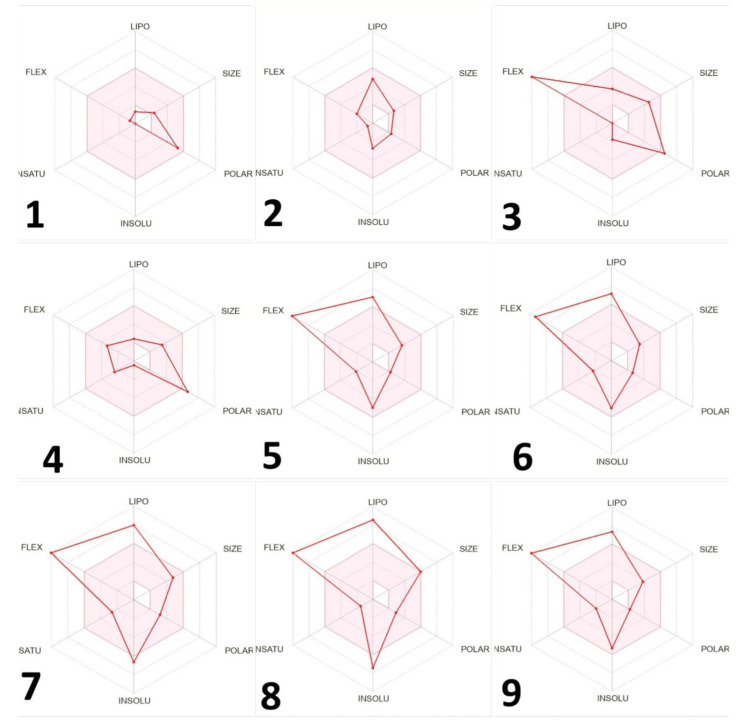
Bioavailability radar and structure of the nine compounds identified in *Silybum marianum* extract: (1) d-Mannose, (2) *N*-methyl-1-adamantaneacetamide, (3) d-Mannitol, 1-decylsulfonyl-(sugar alcohol with sulfur), (4) Desulfosinigrin, (5) 9,12-Octadecadienoic acid, methyl ester, (6) Linoleic acid, (7) Diisooctyl phthalate, (8) 1-Monolinoleoylglycerol trimethylsilyl ether, (9) Mandenol. The pink area indicates the optimal range for each property (lipophilicity: XLOGP3 between 0.7 and +5.0; size: MW between 150 and 500 g/mol; polarity: TPSA between 20 and 130 Å^2^; solubility: log S not higher than 6; saturation: carbon fraction within the SP3 hybridization no less than 0.25; and flexibility: no more than 9 rotatable bonds).

The absorption and distribution parameters of the nine compounds revealed divergent trends between the more polar and the more hydrophobic molecules (Table 5). P-gp inhibition was negligible for most compounds except 1-monolinoleoylglycerol trimethylsilyl ether, which exhibited a significant inhibition of 0.968. Human intestinal absorption (HIA) was high for the more hydrophilic d-mannose (0.899) and d-mannitol (0.938), but poor for hydrophobic 1-monolinoleoylglycerol trimethylsilyl ether (0.001). Similarly, the fraction absorbed at lower doses was high only for the first four most polar compounds, suggesting solubility-limited absorption for the more lipophilic molecules. In terms of distribution, the blood-brain barrier penetration (BBB) was substantial only for N-methyl-1-adamantaneacetamide (0.982) among the tested compounds. Plasma protein binding (PPB) exceeded 95% for hydrophobic compounds including linoleic acid (96.84%) and diisooctyl phthalate (98.39%), while the unbound fraction was correspondingly low. Volume of distribution also trended higher for more lipophilic molecules, such as linoleic acid (2.926 L/kg). Overall, the hydrophobic compounds exhibited poorer absorption but higher plasma binding and volume of distribution versus the more hydrophilic molecules. The absorption and distribution profiles showed advantageous trends for d-mannose and N-methyl-1-adamantaneacetamide, with optimal ADME and toxicity properties. The remaining compounds had some unfavorable parameters, in particular high plasma protein binding, poor aqueous solubility, and potential toxicity issues (Figure 4). This means that d-mannose and N-methyl-1-adamantaneacetamide displayed the most promising overall drug-like properties.

**Figure 5:**
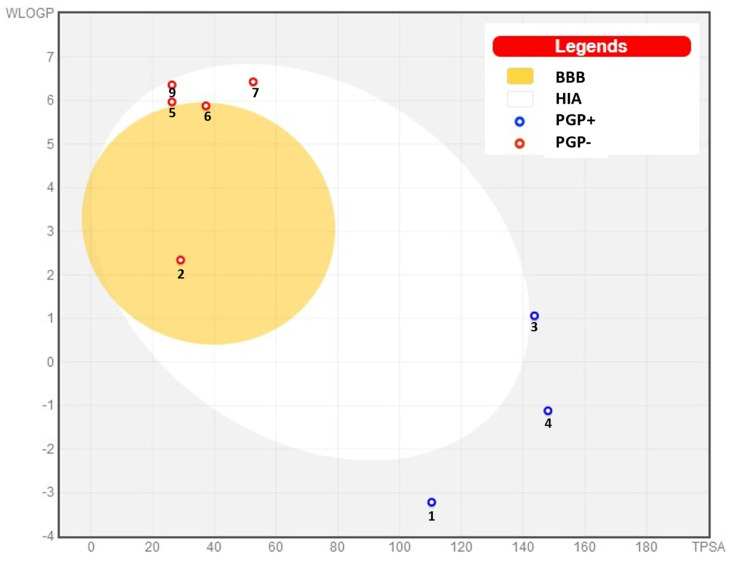
SwissADME BOILED-Egg diagram of bioactive compounds from the *Silybum marianum* seed extract for perceptive evaluation of their passive gastrointestinal absorption (HIA) and blood-brain barrier (BBB) penetration. (1) d-Mannose, (2) N-methyl-1-adamantaneacetamide, (3) d-Mannitol, 1-decylsulfonyl-(sugar alcohol with sulfur), (4) Desulfosinigrin, (5) 9,12-Octadecadienoic acid, methyl ester, (6) Linoleic acid, (7) Diisooctyl phthalate, and (9) Mandenol. Note that molecule no. 8 (1-Monolinoleoylglycerol trimethylsilyl ether) is out of the range presented in this diagram.

A BOILED-Egg diagram (Figure 5) constructed to evaluate passive gastrointestinal absorption (HIA) and brain penetration (BBB) of the nine compounds revealed that compounds **2**, **5**, **6**, **7**, and **9** were P-glycoprotein non-substrates (PGP-), while **1**, **3**, and **4** were PGP substrates (PGP+). Compounds **7** and **9** fell in the white region, suggesting good gastrointestinal absorption, while compounds **2**, **5**, and **6** localized to the yolk, indicating potential BBB penetration. However, compounds **1**, **3**, and **4** were outside the egg, implying limited absorption and BBB penetration. Compound **8** was out of the range of gastrointestinal absorption (HIA) and brain penetration (BBB).

## 3. Discussion

Wound infections are serious infections that contribute to increased mortality and morbidity worldwide, particularly in developing countries [5]. Owing to the emergence of bacterial resistance against common antibiotics, the treatment of wound infections has become more complicated. Therefore, there is an urgent need for safe alternative antibacterial agents. This study included 30 bacterial isolates previously obtained from superficial, infected wounds. Sixty percent of these isolates were assigned to Gram-positive cocci, represented by one species—*S. aureus*. The remaining isolates were identified as Gram-negative bacilli (including *P. aeruginosa*, *K. pneumoniae*, *E. coli*, and *S. maltophilia*). This result is consistent with other studies that report that *S. aureus* is the most frequently isolated bacteria from infected wounds [47,48].

The antibiotic sensitivity tests of the 30 bacterial isolates revealed a high variation among the isolates in antibiotic resistance, ranging from 0.0% to 94.4% of the tested antibiotics (Table 1). Three Gram-negative bacterial isolates (nos. NDL224, NDL225, and NDL2210), identified as *K. pneumoniae*, *E. coli*, and *S. maltophilia,* were considered MDR bacteria because they were resistant to most (88.9–94.4%) of the tested antibiotics belonging to different groups [49]. On the other hand, from Gram-positive bacteria, one isolate of *S. aureus* (no. NDL2220) was resistant to 83.3% of antibiotics; it resisted cefoxitin, benzylpenicillin, and oxacillin, which confirmed that *S. aureus* was a methicillin-resistant *Staphylococcus aureus* (MRSA), which is considered one of the most dangerous bacteria [50]. *S. aureus* was highly sensitive to linezolid, vancomycin, trimethoprim/sulfamethoxazole, and rifampicin, which agrees with other studies [51]. *P. aeruginosa* strains were susceptible to levofloxacin, followed by ciprofloxacin; however, they showed resistance against aztreonam and ceftazidime; this was in agreement with two other reports [52,53]. *E. coli* isolates were highly sensitive to gentamycin and imipenem but presented resistance to ampicillin and ciprofloxacin; these results are consistent with an earlier study by Gomatheswari and Jeyamurugan [54]. *S. maltophilia* was resistant to all antibiotics except trimethoprim/sulfamethoxazole. *K. pneumoniae* strains were very sensitive to amikacin and imipenem but resistant to ampicillin-sulbactam, tetracycline, and ampicillin. These results are consistent with the report by Gomatheswari and Jeyamurugan [54]. Data on the antibiotic resistance of the four above-mentioned isolates were confirmed using VITEK2 Compact_15 for ID&AST (Tables S1 and S2). Primary morphological features of colonies, secondary (automatic) identification using the Biomerieux Vitek 2 System, and molecular analysis of 16S rRNA sequences confirmed the species identification of these MDR isolates (Table S3 and Figure 1).

Our results revealed that *S. marianum* seed extracts exhibited considerable antibacterial activity, especially the ethanol extract. All the isolates were more sensitive to *S. marianum* seed ethanol extract compared to the other extracts (Table 2). The acetone extract exhibited an antibacterial effect against MRSA, but it did not show any effect against Gram-negative bacteria. The acetone extract may contain active constituents with narrow-spectrum activity versus Gram-positive bacteria. The difference in the effect of acetone extract on two types of bacteria may be attributed to the differences in their cell wall structures. Gram-negative organisms are considered to be more resistant due to their outer membrane acting as a barrier to many antibacterial substances [55]. The cold extract showed antibacterial effects only against Gram-negative bacteria. This suggests that the cold extract may contain active constituents with narrow-spectrum activity against Gram-negative bacteria (Table 2). These differences between Gram-positive and Gram-negative bacteria may be related to differences in composition between the two types of bacterial cells. The hot extract did not express any antibacterial activity against Gram-positive and Gram-negative bacteria, which is probably related to the heat-induced decomposition of its active constituents and may also be related to the poor solubility of silymarin (the main active constituent in the seeds of *S. marianum*) [56].

The ethanol extract of *S. marianum* seeds exhibited the maximum inhibition zone against both Gram-positive and Gram-negative bacteria, consistent with the outcomes of previous studies [29,57]. The MIC results (Table 3) showed that the minimum concentrations of the *S. marianum* seed ethanol extract that could inhibit bacterial growth were between 9.375 and 1.172 mg/mL. MBC exceeded four times the MIC, indicating that the ethanol extract had a bacteriostatic effect on the tested bacterial strains.

The antibacterial effects of *S. marianum* seed ethanol extract on the cell ultrastructure of MRSA and *S. maltophilia* were determined by TEM. In contrast to the cells in the control samples, the cells treated with the MIC of the ethanol extract showed abnormal changes in the ultrastructure: damaged cell walls and ruptured cell membranes, which caused the release of the internal contents, plasmolysis, and vacuolization, leading to cell collapse (Figure 2 and Figure 3). Lahlah et al. [23] reported that the antibacterial efficacy of *S. marianum* seed extract was realized through inhibition of RNA and protein synthesis. GC–MS analysis of *S. marianum* seed ethanol extract demonstrated the presence of active compounds with many biological effects reported in the literature (Table 4). The extract contained a variety of compounds with antibacterial properties (linoleic acid, d-mannitol, 1-decylsulfonyl-, ethyl linoleate, d-mannose, 1-monolinoleoylglycerol trimethylsilyl ether, N-methyl-1-adamantaneacetamide, and desulfosinigrin). The most abundant compound was linoleic acid (20%), which is known for its potential antibacterial activity. Greenway and Dyke [58] and Elshobary et al. [12] suggested that linoleic acid probably inhibited growth by increasing the permeability of the bacterial membrane as a result of its surfactant action. Linoleic acid was also stated as a model compound of unsaturated fatty acids that selectively inhibited the FabI enzyme and therefore could stop fatty acid production in bacterial membranes [59]. All nine major components have documented biological activities or medical benefits, including immune-enhancing, antitumor, anticancer, antioxidant, antimicrobial, and anti-inflammatory effects, as mentioned in Table 4. Interestingly, the antibacterial activities of the ethanol extract may be correlated to the synergistic effect of these various compounds. Therefore, *S. marianum* seed ethanol extract can be considered an alternative antibacterial agent against the selected MDR bacteria, consistent with the findings of previous studies [29,57,60].

The potential of a promising drug can be ruined by its limited characteristics of absorption, distribution, metabolism, excretion, and toxicity (ADMET) [61]. A major drawback of drug discovery in clinical trials is the expensive testing of the pharmacokinetic properties. Consequently, in silico methods were used to estimate ADMET parameters to predict whether a specific component of the extract would be a good candidate for drug development. Drug-likeness, as well as the ability to form chemical bonds with protein receptors, is mainly affected by the number of atoms that form H bonds with acceptors and donors. Thus, the results (Table 5) show an acceptable number of donors and acceptors, which clarifies the antimicrobial properties. Our analysis revealed promising property ranges for nine compounds to guide future antimicrobial agent design. Optimal values included molecular weights of 200–600. The hydrogen bond donor and acceptor numbers were within acceptable limits to enable binding to targets. These compounds may form stable complexes and show desired biological activities through favorable hydrogen bonding interactions. The absorption and distribution parameters revealed overall favorable trends for these compounds. P-glycoprotein inhibition was high only for 1-Monolinoleoylglycerol trimethylsilyl ether (0.968), indicating that most compounds are unlikely to cause P-gp mediated drug-drug interactions. Human intestinal absorption (HIA) was high for the more hydrophilic d-mannose (0.899) and d-Mannitol (0.938), meeting the requirement for oral drug efficacy. Plasma protein binding (PPB) fell within acceptable ranges, between 12.5 and 100.9% across the compounds. Parameters related to metabolism, excretion, and toxicity also exhibited drug-like properties. While the more hydrophobic molecules showed lower absorption, their distribution properties were not prohibitive.

In summary, the diverse set of compounds displayed largely promising absorption, distribution, metabolism, excretion, and toxicity profiles. With some physicochemical optimization, particularly for the absorption of the more lipophilic molecules, these favorable ADMET characteristics highlight the potential of these compounds as drug candidates. The first step in the absorption process is the breakdown of the tablet or capsule, followed by the dissolution of the active ingredient. The evaluation of this property early in the drug discovery process is crucial since low solubility hinders effective and complete oral absorption. LogS must range between −4 and 0.5 log mol/L, so our first four compounds have a solubility of −0.017 to −2.564. The rest of the compounds have a solubility assessment of −7.192 due to the oil nature of the compounds. LogP significantly influences membrane permeability and binding to proteins, transporters, and enzymes. LogP ranged from −2.499 to 0.183 for the first 3 compounds, within the optimal 0 to 3 log mol/L range. While the first few compounds exhibited promising absorption properties, the hydrophobic compounds displayed limitations that required optimization through structural modifications.

For a medicine to have a therapeutic effect, it must enter the bloodstream and travel to the site of action. The ability of a medicine to dissolve in body fluid and permeate the biomembrane depends on maintaining a balance between lipophilicity and hydrophilicity. In the early stages of drug discovery, it is crucial to estimate n-octanol/water distribution coefficients at physiological pH (logD7.4). Interpretation of findings: The logarithm of the molar concentration (log mol/L) is used to represent the expected logD7.4 of a chemical. Compounds should be between 1 and 3 log mol/L. It is also equivalent to the accepted score for four compounds. P-glycoprotein inhibitors are membrane proteins that belong to the ABC transporter family. It is also known as MDR1 or 2 ABCB1. Due to the fact that it recognizes xenobiotics with varying structural differences and that they appear to be unrelated, it is arguably the most promiscuous efflux transporter. It is noteworthy that many of these xenobiotics are also CYP3A4 substrates [62,63]. As a result, our compounds show comparable absorption parameter values that were previously observed in recent studies [64,65]. A medication’s apparent efficacy depends on its ability to be absorbed by the human gut. HIA can also serve as a substitute indicator for oral bioavailability since oral bioavailability and intestinal absorption are closely related. To reach the systemic circulation, oral medications must pass through the intestinal cell membranes via passive diffusion, carrier-mediated absorption, or active transport. Often used to assess in vivo drug permeability, human colon adenocarcinoma cell lines (Caco-2) have morphological and functional similarities to the human intestinal epithelium. Caco-2 cell permeability has become a crucial indicator for a prospective medicinal molecule [66]. For our tested sets, our results appear to be in line with accepted wide ranges of metabolism. 

The clearance of a drug can also be considered in addition to its excretion value, which refers to the half-life in terms of predicted CL penetration. For each parameter, we found acceptable ranges. Plasma protein binding (PPB) is one of the main methods of drug uptake and distribution; hence, a medication’s affinity for plasma proteins has a significant impact on how it behaves pharmacologically. During this process, when the drug binds to serum proteins, the free concentration of the drug is at risk, which can have a direct effect on its oral bioavailability. The PPB of a compound is deemed suitable if it has an anticipated value greater than 90%. For 4 of 9 of our compounds, this value was noted, and it appeared not all of our compounds penetrated PPB. The oral bioavailability of our compounds is moderate to acceptable [67]. The chemical basis of biotransformation can be broadly classified as Phase I (oxidative reactions) and Phase II (conjugative reactions) of the drug metabolism process. Two thirds of known drugs are metabolized by the 57 isozymes of the human cytochrome P450 family (phase I enzymes), with isozymes A2, 3A4, 2C9, 2C19, and 2D6 accounting for 80% of this process. Most of these phase I reactions are carried out in the liver by CYPs. Categorized as Category 0 are non-substrates/non-inhibitors; Categorized as Category 1 are substrates/inhibitors. In the range of 0 to 1, the output value represents the probability of being a substrate or an inhibitor. Due to this, we can show 5 compounds with a moderate to good CL and the rest with CLs greater than 5. 

The toxicity of certain substances can also be assessed by using many parameters, such as hERG blockers and H-HT (human hepatotoxic), and our results show a wide range of values, with compound 1 showing the lowest toxicity and no compound showing any carcinogenic toxicity [68,69]. Our compounds have good synthetic acceptability and are easy to synthesize. An oral bioavailability radar based on this revealed mostly favorable trends for d-mannose and N-methyl-1-adamantaneacetamide, with both compounds having properties across ADME and toxicity in the optimal range. The other compounds exhibited some suboptimal parameters, with high plasma protein binding, low aqueous solubility, and concerns regarding toxicity being the most prevalent (Figure 5). In summary, d-mannose and *N*-methyl-1-adamantaneacetamide exhibited the most ideal drug-like characteristics overall, while the other compounds may require chemical modifications to enhance their ADMET properties. Targeted optimization efforts could improve the less favorable absorption, distribution, metabolism, excretion, and toxicity characteristics of these compounds. According to the BOILED-Egg diagram, compounds **7** and **9** had good gastrointestinal absorption, compounds **2**, **5**, and **6** had potential BBB penetration, while compounds **1**, **3**, and **4** imply limited absorption and BBB penetration.

## 4. Materials and Methods

### 4.1. Bacteria Collection

Thirty purified bacterial cultures were obtained during 2021–2022 from the collection of bacterial isolates maintained at the Nile & Delta LAB (NDL), Microbiology Unit (Desouq, Kafr El-Sheikh, Egypt). These isolates were previously collected from superficial infected wounds following the guidelines and standard protocols in accordance with the requirements of the Declaration of Helsinki [70]. The collected purified bacterial cultures were transferred to the Laboratory of Microbiology at the Department of Botany and Microbiology, Faculty of Science, Tanta University. Subsequently, all bacterial cultures were subcultured immediately after collection on nutrition agar, MacConkey agar, mannitol salt agar, and blood agar plates, and then stored at 4 °C until further use [7].

### 4.2. Bacteria Identification

Primarily, the identification of bacterial strains was based on the morphological features of colonies [71]. The MDR bacterial isolates were subjected to secondary (automatic) identification using the Biomerieux Vitek 2 System [72,73] at the Nile & Delta LAB (Nile & Delta LAB, Desouq, Kafr El-Sheikh, Egypt), Microbiology Unit. Complete bacterial strain identification for MDR strains was conducted via molecular analysis of the 16S rRNA gene [74]. Bacterial isolates were grown in autoclaved test tubes containing nutrient broth medium and incubated at 28 °C for 48 h. Broth cultures were transferred to Sigma Scientific Services Co. (El Housarry, 6 of October, Cairo, Egypt). The genomic DNA of the bacterial isolates was extracted using the Gene JET Genomic DNA Purification Kit (Thermo Scientific # k0721). The 16S rRNA fragments were amplified by PCR using Maxima Hot Start PCR Master Mix (Thermo K1051) from Sigma Company of Scientific Services, El Housarry, 6 of October, Cairo, Egypt (https://www.sigmaeg-co.com/, accessed on 14 March 2022). A region of ~1000 bp from the 16S rRNA gene was amplified using two universal primers, namely 27F (5′-AGAGTTTGATCCTGGCTCAG-3′) and 1492R (5′-GGTTACCTTGTTACGACTT-3′). The PCR program consisted of one cycle of DNA initial denaturation at 95 °C (10 min), 35 cycles of 95 °C (30 s), 65 °C (1 min), and 72 °C (1 min and 30 s), plus one additional cycle of a final chain elongation at 72 °C (10 min). Finally, sequencing was accomplished on the PCR product of GATC Company by means of an ABI 3730xl DNA sequencer using forward and reverse primers. Sequences were further analyzed using the Basic Local Alignment Search Tool (BLAST) from the National Center of Biotechnology Information web site (NCBI, http://www.ncbi.nlm.nih.gov/blast/, accessed on 15 April 2022), and the closely similar species were identified by detection of the scores of percent homology. The sequences were aligned using Clustal W in MEGA 11 with the default settings [75]. Furthermore, MEGA 11 software was used to create a dendrogram using the neighbor-joining technique [76] and the parameter distance [77].

### 4.3. Preparation of Bacterial Suspension

The surface viable counting technique described by Collee et al. [78] was used to calculate the average number of viable organisms per milliliter of the stock suspension (the direct colony suspension technique was utilized to prepare the bacterial suspensions) from the fresh plates of each bacterial culture. A few single colonies were transferred by a sterile needle into screw-capped tubes containing 10 mL of 0.9% sterile normal saline (stock suspension). Each stock suspension was diluted by transferring 1 mL from each stock tube to the screw-capped tubes containing 9 mL of normal saline (vortexing was performed for shaking tubes). Then, 0.02 mL from each diluted tube was added dropwise using an automated micropipette to the nutrient agar surface. After drying at room temperature for two hours, the plates were incubated for 24 h at 37 °C. Following incubation, the number of visible colonies in each plate was counted and multiplied by 50 and the dilution factor to obtain the number of viable bacterial colonies per milliliter, which was expressed as a colony-forming unit per mL (CFU/mL). Each bacterial suspension was diluted to reach a cell count of ~106 CFU/mL.

### 4.4. Antibiotic Susceptibility Test

An antibiotic susceptibility test of the fresh bacterial suspension of each isolate (10^6^ CFU/mL) was performed by means of a disc diffusion (Kirby–Bauer) method [79]. Discs with 18 antibiotics from 10 different classes were used to test the susceptibility of Gram-negative bacteria, while discs with 12 antibiotics from 10 different classes were used to test the sensitivity of Gram-positive bacteria (Table 6). The results for the most MDR bacteria were confirmed by automatic antibiotic susceptibility tests carried out using VITEK2 Compact_15 for ID&AST at Nile & Delta LAB, Microbiology Unit. The results of manual and automatic antibiotic susceptibility tests were analyzed according to the diameter clear zone (mm) and MIC Break points for each bacterial isolate of Clinical and Laboratory Standards Institute M100 (CLSI) [80] to determine the resistance and sensitivity of bacteria to antibiotics.

### 4.5. Plant Extract Preparation

*S. marianum* seeds were collected in June 2022, after blooming, from a field belonging to the Sakha Horticulture Research Station (Kafr El-Sheikh governorate, Egypt). The plants designated for seed collection were identified by Dr. Awad Y. Shala (Senior Researcher at the Medicinal and Aromatic Plants Research Department, Horticulture Research Institute, Agricultural Research Center, Giza, Egypt) using available taxonomic keys. The seeds were rinsed under running tap water to remove any dust and foreign particles, and then washed with distilled water. The seeds were left to dry at room temperature, shaded from the sunlight. Then, they were ground to a fine powder using an electric grinder, stored in clean, dark, airtight vessels, and subsequently subjected to extraction using different solvents (ethanol, acetone, and cold and hot water). Plant extraction was based on the method of Abu-Al-Basal [81], with slight modifications.

#### 4.5.1. Ethanol Extract

In a 500 mL sterile conical flask, 50 g of dried milk thistle seed powder was meticulously extracted with 200 mL of 95% ethanol. The flask was closed using a cotton plug and aluminum foil and kept on a rotary shaker for 48 h at room temperature. The extract was filtered using a sterile cloth sheet. The filtrate was centrifuged at 3000 rpm for 15 min, then placed in sterile Petri dishes and allowed to dry under an air fan at room temperature to remove residual solvents. The obtained crude extract was weighed in grams and kept in sterile, dark bottles at 4 °C until further use [81]. Then, 600 mg/mL of plant extract was prepared using dimethyl sulfoxide (DMSO).

#### 4.5.2. Acetone Extract

The acetone extract was obtained using the same protocol as the ethanol extract, but in the first extraction step, 50 g of dried milk thistle seed powder was extracted with 200 mL of acetone instead of 95% ethanol. 

#### 4.5.3. Cold Aqueous Extract

Dried milk thistle seed powder (30 g) was added to 300 mL of sterile distilled water in a 500 mL sterile conical flask. The flask was then plugged with cotton wool and placed on a rotary shaker at room temperature for 24 h. The extract was filtered using a sterile cloth sheet. The filtrate was centrifuged at 3000 rpm for 15 min, placed on sterile Petri dishes, and evaporated in a water bath at 75 °C under an air fan for drying. The resulting crude extract was weighed and stored in a sterile, dark bottle at 4 °C until further use [82]. Then, 600 mg/mL of plant extract were prepared using DMSO.

#### 4.5.4. Hot Aqueous Extract

The hot aqueous extract of *S. marianum* seeds was obtained using the same protocol as the cold extract, except that the mixture of plant powder and distilled water was left until boiling for 30 min before being transferred to a shaker [81,83].

### 4.6. In Vitro Antibacterial Activity of Plant Extracts against MDR Bacteria

The investigation of the antibacterial activity of plant extracts was performed using the agar-well diffusion technique [53,84]. Each of the fresh bacterial suspensions (106 CFU/mL; 0.1 mL) was spread homogeneously on the solidified surface of Mueller–Hinton agar plates (Oxoid Ltd., London, UK) using a sterile cotton swab, and the plates were left to dry for 15 min. A sterile cork borer was used to create four 6 mm-diameter wells in agar on each plate, allowing for the application of four types of extracts: ethanol, acetone, cold water, and hot water. Then, 0.1 mL of each plant extract (600 mg/mL) was distributed into the wells using a micropipette. The plates were placed in a refrigerator for 1 h at 4 °C to allow the plant extracts to diffuse into the media. DMSO was used as a negative control. Then, the plates were incubated at 37 °C for 24 h, and the clear zone diameters were measured in millimeters. The above-mentioned steps were repeated three times.

### 4.7. Determination of Minimum Inhibitory Concentrations and Minimum Bactericidal Concentrations

The minimum inhibitory concentration (MIC) is the lowest concentration of extract that is required to inhibit bacterial growth. The MICs were determined using the colorimetric broth microdilution method. A 96-well microtiter plate was used. Serial dilutions of each plant extract were prepared using DMSO to achieve a final concentration of 150 mg/mL. In each well, 50 μL of nutrient broth was added, and 50 μL of plant extracts (150 mg/mL) were placed in the first well. Using a two-fold micropipette, serial dilutions were conducted from the first to the twelfth well. The resulting concentrations were between 75 and 0.0366 mg/mL, and 10 μL of bacterial suspensions (106 CFU/mL) were added to all wells except the negative control, which contained only nutrient broth. The positive control wells contained nutrient broth with an inoculum. The microtiter plates were incubated for 18 h at 37 °C, and 0.01% 2,3,5-triphenyl tetrazolium chloride (TTC) was used as a bacterial growth indicator. After incubation, 50 of μL TTC solution was added to each well, and the plates were further incubated at 37 °C for 2 h. The MIC was detected at the lowest plant extract concentration that inhibited bacterial growth and prevented TTC color change. Bacterial growth was determined by the color change of TTC from clear to red through reduction. The positive control turned red, while the negative control remained clear (did not show a color change of TTC) [53,85,86]. The minimum bactericidal concentration (MBC) is the lowest concentration of a plant extract that is required to kill bacterial cells where there is no bacterial growth visible. The MBC values were determined from broth-dilution MIC tests. A loopful of the contents from each well was streaked onto a solidified nutrient agar plate. After 24 h at 37 °C, the growth of bacteria was observed. If the MBC is not greater than four times the MIC, the antibacterial agent is considered bactericidal [87].

### 4.8. Effect of S. marianum Seed Ethanol Extract on Bacterial Strain Cell Ultrastructure via Transmission Electron Microscopy

The antibacterial effects of *S. marianum* seed ethanol extract on methicillin-resistant *Staphylococcus aureus* (MRSA) and *Stenotrophomonas maltophilia* were determined using transmission electron microscopy (TEM), as both of these isolates showed higher sensitivity to ethanol extract compared to other isolates. Fresh bacterial suspensions (106 CFU/mL) of each selected isolate were treated with the previously recorded MICs of the plant extract, and the untreated bacterial cells without the addition of the extract constituted a control. These samples were incubated overnight at 37 °C on a shaking incubator at 60 rpm, and the specimens were prepared for examination [88]. Finally, alterations in the bacterial ultrastructure were photographed using TEM (JEM-1400 Plus, JEOL, Japan) at the Electron Microscope Unit, Faculty of Science, Alexandria University (Alexandria, Egypt).

### 4.9. Gas Chromatography–Mass Spectroscopy

The *S. marianum* seed ethanol extract, which exhibits potent antibacterial potential against MDR bacteria, was analyzed by gas chromatography–mass spectroscopy (GC–MS) to detect its main components possessing pharmacological activities, especially antibacterial activity. GC–MS analysis was performed at the Scientific Research Center and Measurement, Tanta University, using the Perkin–Elmer model: Clarus 580/560S with Elite-5MS fused silica capillary column (30 m × 0.25 mm × 0.25 µm film thickness). Helium gas (99.9995%) was used as a carrier gas at a flow rate of 1 mL/min. The split ratio was adjusted to 1:20. The solvent delay was 6 min, the injection volume was 1 μL, and the scan range was 50–620 Da. The oven temperature was started at 80 °C (maintained for 7 min), increased at the rate of 10 °C/min to reach 140 °C (maintained at 140 °C for 1 min), increased at a rate of 10 °C/min to reach 200 °C (maintained at 200 °C for 2 min), and increased at a rate of 5 °C/min to reach a final temperature of 280 °C (maintained at 280 °C for 10 min). The total running time of GC was 48 min. The separated peaks of the compounds were identified using the databases of the NIST08, WILEY8, and FAME libraries. The mass spectrum of each unknown compound was compared with that of the known compounds recorded in the software database libraries to determine the name, structure, and molecular weight of each compound.

### 4.10. Molecule Retrieval

The main components detected by GC-MS were retrieved from the PubChem database (https://pubchem.ncbi.nlm.nih.gov, accessed on 26 April 2023) [89] as canonical SMILES strings and saved separately.

### 4.11. The Pharmacokinetic and ADMET Prediction

SMILES strings were submitted into ADMETlab2.0 (https://admetmesh.scbdd.com, accessed on 26 April 2023) [61] and then screened using the SwissADME web server (http://www.swissadme.ch, accessed on 26 April 2023) [90] for evaluation and Boiled egg chart detection.

### 4.12. Statistical Analysis

IBM SPSS version 27 was used for statistical analysis. The mean and standard deviation (SD) of three readings were used to represent the results. The data were statistically analyzed to determine the significant differences among the different treatments using one-way analysis of variance (ANOVA). The significance level was set at α = 0.05. Post-hoc tests were performed to reveal statistically significant differences among the experimental groups.

## 5. Conclusions

According to this study, the ethanol extract from *S. marianum* seed was effective anti-bacterial (MIC and MBC values indicated a bacteriostatic effect), and its components had potential drug-likeness properties, bioavailability with low toxicity, and accepted ADMET values. Among 30 bacterial isolates from superficial, infected wounds, four were MDR, namely *S. aureus* AAE, *S. maltophilia* AAE, *K. pneumoniae* AAE, and *E. coli* AAE. Their 16S rRNA sequences were deposited in GenBank under accession numbers LC764399, LC764400, LC764401, and LC764402, respectively. *S. marianum* seed ethanol extract showed antibacterial effects by denaturing and deforming the ultrastructure of MRSA and *S. maltophilia*. GC–MS analysis of this extract revealed numerous bioactive components that may be responsible for its antibacterial activity. However, we only used four MDR strains to test the antibacterial effect of *S. marianum* seed extracts. Conducting a large-scale screening of MDR strains would allow us to obtain more data and explore the full potential of the extracts. In the subsequent research, we would be able to modify pure compounds that could be used as a promising alternative antibacterial agent to help control bacterial resistance in wound infections. Further testing is required, including fractionation, purification of the active compounds, and in vitro testing of the antimicrobial activity of the purified active compounds against a broad range of bacterial strains. These bioactive compounds need to be tested in vivo on infection-modeling animals to determine their clinical significance and establish a reliable correlation between in vitro and in vivo results. Structural modification of compounds can improve their pharmacokinetics, pharmacodynamics, and structure-activity relationships. Future research will include studying the synergies between pure compounds and antibiotics, evaluating the efficacy of plant extracts and active component–antibiotic combinations in vitro and in vivo, and developing wound dressing fibers infused with active purified compounds to combat wound infections.

## Figures and Tables

**Table 1:** Results of species identification and screening for antibiotic resistance (using the disc diffusion method) were obtained for 30 bacterial isolates from superficial, infected wounds.

Gram Stain Test	Bacterial Isolate Code Number	Species *	Number (and Percentage) of Tested Antibiotics to Which the Isolate Showed:	
			Resistance	Susceptibility
Gram-negative bacilli	NDL221	*E. coli*	8 (44.4%)	10 (55.5%)
	NDL222	*P. aeruginosa*	10 (55.5%)	8 (44.4%)
	NDL223	*K. pneumoniae*	12 (66.7%)	6 (33.3%)
	**NDL224**	***K. pneumoniae***	**16 (88.9%)**	**2 (11.1%)**
	**NDL225**	***E. coli***	**17 (94.4%)**	**1 (5.5%)**
	NDL226	*E. coli*	11 (61.1%)	7 (38.9%)
	NDL227	*P. aeruginosa*	10 (55.5%)	8 (44.4%)
	NDL228	*P. aeruginosa*	6 (33.3%)	12 (66.7%)
	NDL229	*P. aeruginosa*	13 (72.2%)	5 (27.8%)
	**NDL2210**	***S. maltophilia***	**17 (94.4%)**	**1 (5.5%)**
	NDL2211	*P. aeruginosa*	7 (38.9%)	11 (61.1%)
	NDL2212	*P. aeruginosa*	5 (27.8%)	13 (72.2%)
Gram-positive cocci	NDL2213	*S. aureus*	7 (58.3%)	5 (41.7%)
	NDL2214	*S. aureus*	4 (33.3%)	8 (66.7%)
	NDL2215	*S. aureus*	0 (0.0%)	12 (100.0%)
	NDL2216	*S. aureus*	8 (66.7%)	4 (33.3%)
	NDL2217	*S. aureus*	1 (8.3%)	11 (91.7%)
	NDL2218	*S. aureus*	2 (16.7%)	10 (83.3%)
	NDL2219	*S. aureus*	0 (0.0%)	12 (100.0%)
	**NDL2220**	***S. aureus***	**10 (83.3%)**	**2 (16.7%)**
	NDL2221	*S. aureus*	4 (33.3%)	8 (66.7%)
	NDL2222	*S. aureus*	4 (33.3%)	8 (66.7%)
	NDL2223	*S. aureus*	9 (75.0%)	3 (25.0%)
	NDL2224	*S. aureus*	7 (58.3%)	5 (41.7%)
	NDL2225	*S. aureus*	5 (41.7%)	7 (58.3%)
	NDL2226	*S. aureus*	0 (0.0%)	12 (100.0%)
	NDL2227	*S. aureus*	3 (25.0%)	9 (75.0%)
	NDL2228	*S. aureus*	0 (0.0%)	12 (100.0%)
	NDL2229	*S. aureus*	6 (50.0%)	6 (50.0%)
	NDL2230	*S. aureus*	2 (16.7%)	10 (83.3%)

* Full species names: Klebsiella pneumoniae, Escherichia coli, Stenotrophomonas maltophilia, Staphylococcus aureus, and Pseudomonas aeruginosa. MDR bacterial isolates are indicated in bold.

**Table 2:** Antibacterial activity of the various extracts of *S. marianum* seeds at a concentration of 600 mg/mL against MDR bacteria. The values provided for each tested species represent the mean inhibition zone (mm) ± standard deviation of three replicates. The *p* and F values represent the results of statistical analysis using one-way analysis of variance (ANOVA).

Extracts of *S. marianum*	Inhibition Zone (mm) Recorded in Tests with:			
	MRSA	*S. maltophilia*	*K. pneumoniae*	*E. coli*
Cold water	0.00 ± 0.00 ^c#^	20.00 ± 2.00 ^b^	11.67 ± 1.15 ^b^	14.33 ± 0.58 ^b^
Hot water	0.00 ± 0.00 ^c^	0.00 ± 0.00 ^c^	0.00 ± 0.00 ^c^	0.00 ± 0.00 ^c^
Ethanol	33.67 ± 1.15 ^a^	34.33 ± 1.15 ^a^	25.00 ± 2.00 ^a^	21.00 ± 2.00 ^a^
Acetone	25.33 ± 1.53 ^b^	0.00 ± 0.00 ^c^	0.00 ± 0.00 ^c^	0.00 ± 0.00 ^c^
DMSO (negative control)	0.00 ± 0.00 ^c^	0.00 ± 0.00 ^c^	0.00 ± 0.00 ^c^	0.00 ± 0.00 ^c^
Total mean	11.80 ± 15.22	10.87 ± 14.58	7.33 ± 10.31	7.07 ± 9.25
*p* value	0.0001 ***	0.0001 ***	0.0001 ***	0.0001 ***
F value	1103.545	694.938	346.094	343.346

^#^ Different lowercase letters in the same column indicate a significant difference, according to post-hoc tests. *** Highly significant result (*p* ≤ 0.0001).

**Table 3:** The minimum inhibitory concentration (MIC) and minimum bactericidal concentration (MBC) of the ethanol extract of *S. marianum* seeds were tested on four species of bacteria.

Tested Bacteria	MIC (mg/mL)	MBC (mg/mL)
MRSA	2.344	18.750
*S. maltophilia*	1.172	9.375
*K. pneumoniae*	2.344	37.500
*E. coli*	9.375	75.000

**Table 4:** Active components of the *S. marianum* seed ethanol extract were identified by means of GC-MS. For each compound, the peak area percentage and biological activity reported in the literature are provided.

Compound Name	Area %	Activity	References
d-Mannose	4.332	Essential food supplement for human health; beneficial effects on the immune system; treatment of urinary tract infections; antitumor agent; beneficial effects against metabolic syndrome; treatment of diabetes and intestinal diseases; and other biological activities	[30,31,32,33]
N-methyl-1-adamantaneacetamide	1.876	Antioxidant and antimicrobial activity	[34]
d-Mannitol, 1-decylsulfonyl-(sugar alcohol with sulfur)	14.839	Anticancer and antimicrobial activities	[35]
Desulfosinigrin	0.83	Antibacterial and antioxidant activities	[12,36,37]
9,12-Octadecadienoic acid, methyl ester, (*E*,*E*)-(linolelaidic acid, methyl ester), or (methyl linolelaidate)	0.616	Antioxidant, antimicrobial, surfactant, hepatoprotective, antihistaminic, hypocholesterolemic, and antieczemic activities	[35,38,39,40]
9,12-Octadecadienoic acid (*Z*,*Z*)-(linoleic acid)	20	Antimicrobial, anti-inflammatory, and antioxidant activities; decreases the rate of developing coronary heart disease; inhibits human breast cancer MCF-7 cells; prevents atherosclerosis, cancer, and hypertension; and improves immune function	[11,41,42,43]
1,2-Benzenedicarboxylic acid, diisooctyl ester (diisooctyl phthalate)	3.276	Antimicrobial and antifouling agents	[35]
1-Monolinoleoylglycerol trimethylsilyl ether	3.097	Antimicrobial, antioxidant, anti-inflammatory, antiarthritic, antiasthma, and diuretic activities	[35]
Linoleic acid ethyl ester (ethyl linoleate) (mandenol)	9.596	Antimicrobial and anti-inflammatory properties of wound healing; effective anti-acne agents used in cosmetics and skin care	[44,45,46]

**Table 5:** Physicochemical and ADMET properties of the nine compounds identified in *Silybum marianum* seed extract: (1) d-Mannose, (2) N-methyl-1-adamantaneacetamide, (3) d-Mannitol, 1-decylsulfonyl-(sugar alcohol with sulfur), (4) Desulfosinigrin, (5) 9,12-Octadecadienoic acid, methyl ester, (6) Linoleic acid, (7) Diisooctyl phthalate, (8) 1-Monolinoleoylglycerol trimethylsilyl ether, and (9) Mandenol. Full data on physiological and ADMET properties with explanations of each parameter are provided in Table S6.

	1	2	3	4	5	6	7	8	9
**Physicochemical Properties**									
Formula	C_6_H_12_O_6_	C_13_H_21_NO	C_16_H_34_O_7_S	C_10_H_17_NO_6_S	C_19_H_34_O_2_	C_18_H_32_O_2_	C_24_H_38_O_4_	C_27_H_54_O_4_Si_2_	C_20_H_36_O_2_
MW (g/mol)	180.16	207.31	370.5	279.31	294.47	280.45	390.56	498.89	308.5
#Heavy atoms	12	15	24	18	21	20	28	33	22
Fraction Csp3	1	0.92	1	0.7	0.74	0.72	0.67	0.81	0.75
#Rotatable bonds	1	3	15	5	15	14	16	22	16
#H-bond acceptors	6	1	7	7	2	2	4	4	2
#H-bond donors	5	1	5	5	0	1	0	0	0
LogS (log mol/L)	−0.017	−2.564	−2.116	−0.517	−6.465	−5.23	−7.04	−7.192	−6.596
LogD (log mol/L)	−2.139	2.709	1.038	−0.695	4.646	3.58	5.345	6.399	4.803
LogP (log mol/L)	−2.499	2.301	0.183	−0.76	6.992	6.652	7.494	8.363	7.217
**Absorption parameters**									
Pgp-inh	0.001	0.035	0.003	0.003	0.001	0	0.968	0.258	0.001
Pgp-sub	0.098	0.001	0.024	0.001	0.028	0.002	0	0.005	0.013
HIA	0.899	0.006	0.938	0.912	0.007	0.01	0.001	0.005	0.003
F(20%)	0.054	0.002	0.996	0.728	0.008	0.009	0.988	0.008	0.008
F (30%)	0.944	0.002	0.972	0.997	0.775	0.549	0.956	0.033	0.729
Caco-2 (log cm/s)	−5.318	−4.62	−5.778	−5.598	−4.551	−4.733	−4.655	−4.811	−4.526
**Distribution parameters**									
BBB	0.48	0.982	0.261	0.569	0.245	0.196	0.013	0.001	0.119
PPB %	12.50%	59.27%	74.81%	45.91%	96.84%	98.39%	97.63%	100.90%	97.29%
VDss (L/kg)	0.395	0.879	0.616	0.401	2.926	0.626	1.445	2.796	2.707
**Metabolism parameters**									
CYP1A2-inh	0.01	0.159	0.016	0.02	0.941	0.235	0.134	0.424	0.939
CYP1A2-sub	0.046	0.314	0.092	0.031	0.179	0.171	0.178	0.605	0.166
CYP2C19-inh	0.01	0.815	0.006	0.017	0.569	0.086	0.699	0.435	0.601
CYP2C19-sub	0.15	0.138	0.226	0.059	0.064	0.066	0.06	0.488	0.058
CYP2C9-inh	0.001	0.506	0.001	0.002	0.6	0.43	0.36	0.66	0.637
CYP2C9-sub	0.16	0.613	0.693	0.584	0.947	0.988	0.878	0.905	0.937
CYP2D6-inh	0.002	0.501	0.001	0.001	0.167	0.006	0.116	0.054	0.337
CYP2D6-sub	0.133	0.295	0.028	0.129	0.139	0.086	0.022	0.262	0.098
CYP3A4-inh	0.004	0.435	0.007	0.009	0.692	0.085	0.254	0.678	0.608
CYP3A4-sub	0.01	0.168	0.027	0.019	0.065	0.019	0.088	0.096	0.068
**Excretion parameters**									
CL (mL/min/kg)	1.474	9.954	4.437	1.449	7.742	3.327	9.241	3.424	7.094
**Toxicity parameters**									
hERG	0.039	0.007	0.263	0.018	0.1	0.009	0.18	0.158	0.104
H-HT	0.046	0.689	0.023	0.074	0.011	0.013	0.003	0.01	0.004
Carcinogenicity	0.013	0.64	0.012	0.572	0.467	0.153	0.334	0.258	0.208
**Medicinal Chemistry**									
Bioavailability Score	0.55	0.55	0.55	0.55	0.55	0.85	0.55	0.55	0.55
Synthetic Accessibility	4.08	3.66	5.37	5.14	3.18	3.1	3.41	5.93	3.34

**Table 6:** The antibiotics used in the sensitivity screening of the bacterial isolates.

Antibiotic Class	Antibiotic	Concentration μg/disc Potency	Chosen for Tests with:	
			Gram-Negative Bacteria?	Gram-Positive Bacteria?
1. Ansamycins	Rifampicin (RIF)	5	no	yes
2. Aminoglycosides	Gentamicin (CN)	10	yes	yes
	Amikacin (AK)	30	yes	no
	Tobramycin (TOB)	10	yes	no
3. Carbapenems	Imipenem (IPM)	10	yes	no
	Meropenem (MEM)	10	yes	no
4. Cephalosporins	Cefepime (CPM)	30	yes	no
	Cefotaxime (CTX)	30	yes	no
	Cefoxitin (FOX)	30	yes	yes
	Ceftazidime (CAZ)	30	yes	no
5. Folate pathway antagonists	Trimethoprim/Sulfamethoxazole (STX)	1.25/23.75	yes	yes
6. Fluoroquinolones	Ciprofloxacin (CIP)	5	yes	yes
	Levofloxacin (lE)	5	yes	yes
7. Macrolide	Azithromycin (AZM)	15	yes	yes
	Erythromycin (E)	15	no	yes
8. Beta-lactam combination agents	Amoxicillin/Clavulanic Acid (AMC)	20/10	yes	no
	Piperacillin/Tazobactam (PIT)	100/10	yes	no
9. Monobactam	Aztreonam (AT)	30	yes	no
10. Phenicols	Chloramphenicol (C)	30	yes	yes
11. Tetracyclines	Tetracycline (TE)	30	yes	yes
12. Lincosamides	Clindmycin (CD)	2	no	yes
13. Oxazolidinone	Linzolid (LZD)	30	no	yes

## Data Availability

Data are contained within the article and Supplementary Materials.

## Supplementary Materials

The following supporting information can be downloaded at: https://www.mdpi.com/article/10.3390/molecules29010064/s1. Table S1: Antibiotic efficiency against three selected isolates of Gram-negative bacteria determined by means of Vitek 2 Compact-15; Table S2: Antibiotic efficiency against *S. aureus* determined by means of Vitek 2 Compact-15; Table S3: Biochemical characterization of multi-drug-resistant bacterial isolates using Biomerieux Vitek; Table S4: Active components of *S. marianum* seed ethanol extract identified by means of GC-MS. For each compound, retention time (RT), peak area percentage, molecular formula (MF), and molecular weight (MW) Table S5: Identified SMILES strings for the nine phytochemical compounds from *Silybum marianum* seed extract; Table S6: ADMET properties of the nine compounds identified in *Silybum marianum* seed extract.
