# Supplementary material for: Effects of Silybum marianum L. Seed Extracts on Multi Drug Resistant (MDR) Bacteria
Source: Molecules. 2023 Dec 21;29(1):64. doi: 10.3390/molecules29010064 (PMC10779956; doi:10.3390/molecules29010064)
Supplement: Supplementary file 1 [file molecules-29-00064-s001.zip › molecules-2737888-supplementary.pdf]

Supplementary Materials

Effects of *Silybum marianum* L. Seed Extracts on Multi Drug Resistant (MDR) Bacteria

Shimaa El-Sapagh [1], Nanis G. Allam [1], Mohamed Nour El-Dein El-Sayed [2], Asmaa Ahmed El-Hefnawy [1,*], Grażyna Korbecka-Glinka [3,*] and Awad Y. Shala [4]

[1] Botany and Microbiology Department, Faculty of Science Tanta University, Tanta 31527, Egypt; shimaa.elsapagh@gmail.com (S.E.-S.)
[2] Soil, Water and Environment Research Institute, Agricultural Research Station, Sakha, Kafr El-Sheikh 33717, Egypt; drmohamednour1961@gmail.com
[3] Department of Plant Breeding and Biotechnology, Institute of Soil Science and Plant Cultivation—State Research Institute, Czartoryskich 8, 24-100 Puławy, Poland
[4] Medicinal and Aromatic Plants Research Department, Horticulture Research Institute, Agricultural Research Center (ARC), Giza 12619, Egypt; awad.shala@yahoo.com
* Correspondence: asmaaahmed2266adelsalam@gmail.com (A.A.E.-H.); gkorbecka@iung.pulawy.pl (G.K.-G.)

Table S1. Antibiotic efficiency against three selected isolates of Gram-negative bacteria *Stenotrophomonas maltophilia, Klebsiella pneumoniae, Escherichia coli* determined by means of Vitek 2 Compact-15.

**Antibiotic**	**Minimum inhibitory concentration (mg/L)**		
	S. maltophilia	***K. pneumoniae***	***E. coli***
Extended-spectrum β-lactamase (ESBL)	Not-detected	NEG	NEG
Ampicillin	≥32 ^{R*}	≥32 ^{R}	≥32 ^{R}
Ampicillin/sulbactam	≥32 ^{R}	≥32 ^{R}	≥32 ^{R}
Amoxicillin/clavulanic acid	≥32 ^{R}	≥32 ^{R}	≥32 ^{R}
Piperacillin/tazobactam	≥128 ^{R}	≥128 ^{R}	≥128 ^{R}
Cefazolin	≥64 ^{R}	≥64 ^{R}	≥64 ^{R}
Cefoxitin	≥64 ^{R}	≥64 ^{R}	≥64 ^{R}
Ceftazidime	≥64 ^{R}	≥64 ^{R}	≥64 ^{R}
Ceftriaxone	≥64 ^{R}	≥64 ^{R}	≥64 ^{R}
Cefepime	≥64 ^{R}	≥64 ^{R}	≥64 ^{R}
Aztreonam	≥64 ^{R}	≥64 ^{R}	≥64 ^{R}
Ertapenem	≥8 ^{R}	≥8 ^{R}	≥8 ^{R}
Imipenem	≥16 ^{R}	≥16 ^{R}	≥16 ^{R}
Meropenem	≥16 ^{R}	≥16 ^{R}	≥16 ^{R}
Amikacin	≥64 ^{R}	16 ^{S}	≥64 ^{R}
Gentamicin	≥16 ^{R}	≥16 ^{R}	4 ^{S}
Tobramycin	≥16 ^{R}	≥16 ^{R}	≥16 ^{R}
Ciprofloxacin	≥4 ^{R}	≥4 ^{R}	≥4 ^{R}
Levofloxacin	≥8 ^{R}	≥8 ^{R}	≥8 ^{R}
Tetracycline	≥16 ^{R}	≥16 ^{R}	≥16 ^{R}
Nitrofurantoin	≥512 ^{R}	64 ^{I}	≥512 ^{R}
Trimethoprim/sulfamethoxazole	40 ^{S}	≥320 ^{R}	≥320 ^{R}

* Interpretation of the result indicated in the superscript: S-sensitive; R: resistant, I: intermediate. NEG: negative (the isolate was sensitive to β-lactam class)

Table S2. Antibiotic efficiency against *S. aureus* determined by means of Vitek 2 Compact-15.

**Antibiotics**	**Minimum inhibitory concentration (mg/L)**	**Interpretation***
Cefoxitin screen	POS	
Benzylpenicillin	≥0.5	R
Oxacillin	≥4	R
Gentamicin	≥16	R
Ciprofloxacin	≤0.5	S
Moxifloxacin	≤0.25	S
Inducible clindamycin resistance	POS	
Erythromycin	≥8	R
Clindamycin	≥4	R
Linezolid	2	S
Teicoplanin	≤0.5	S
Vancomycin	≤0.5	S
Tetracycline	≥16	R
Fusidic acid	8	R
Rifampicin	≤0.5	S
Trimethoprim/sulfamethoxazole	≤10	S

* Interpretation of the results: S-sensitive; R: resistant, POS: positive (*S. aureus* was resistant to Cefoxitin)

Table S3. Biochemical characterization of four multi drug resistant bacterial isolates using Biomerieux Vitek. The percentages (%) provided next to species names indicate probability of their identification.

Test (Mnemonic)		*K.pneumoniae* (98%) (isolate no. NDL224)	*E. coli* (99%) (isolate no. NDL225)	*S. maltophilia* (99%) (isolate no. NDL2210)	*S. aureus* (98%) (isolate no. NDL2220)
Ala-Phe-Pro-Arylamidase	**APPA**	-	-	+	-
Adonitol	**ADO**	+	-	-	
L-Pyrrolydonyl-Arylamidaes	**PyrA**	+	-	-	+
L-Arabitol	**lARL**	-	-	-	
D-Cellobiose	**dCEL**	+	-	-	
Beta-Galactosidase	**BGAL**	+	+	-	-
H_2S production	**H_2S**	-	-	-	
Beta-N-Acetyl-Glucosaminidase	**BANG**	-	-	-	
Glutamyl Arylamidase pNA	**AGLTp**	-	-	-	
D-Glucose	**dGLU**	+	+	-	
Gamma-Glutamyl-Transferase	**GGT**	+	-	+	
Fermentation/ Glucose	**OFF**	+	+	-	
Beta-Glucosidase	**BGLU**	+	-	+	
D-Maltose	**dMAL**	+	+	-	+
D-Mannitol	**dMAN**	+	+	-	+
D-Mannose	**dMNE**	+	+	-	+
Beta-Xylosidase	**BXYL**	+	-	-	
Beta-Alnine arylamidase pNA	**BAlap**	-	-	-	
L-Proline Arylamidase	**ProA**	-	-	+	-
Lipase	**LIP**	-	-	+	
Palatinose	**PLE**	+	-	-	
Tyrosine Arylamidase	**TyrA**	+	-	-	-
Urease	**URE**	-	-	-	+
D-Sorbitol	**dSOR**	+	+	-	-
Saccharose/Sucrose	**SAC**	+	+	-	+
D-Tagatose	**dTAG**	-	-	-	
D-Trehalose	**dTRE**	+	+	-	+
Citrate (Sodium)	**CIT**	+	-	+	
Malonate	**MNT**	+	-	-	
5-Keto-D-Glucnate	**5KG**	-	-	-	
L-Lactate alkalinisation	**lLATk**	+	+	+	+
Alpha-Glucosidase	**AGLU**	-	-	+	+

Succinate alkalinisation	**SUCT**	-	+	+	
Beta-N-Acetyl-Galactosaminidase	**NAGA**	-	-	-	
Alpha-Galactosidase	**AGAL**	+	+	+	-
Phosphatase	**PHOS**	+	-	+	+
Glycine Arylamidase	**GlyA**	+	-	-	
Ornithine decarboxylase	**ODC**	-	+	-	
Lysine decarboxylase	**LDC**	+	+	-	
L-Histidine assimilation	**lHlSa**	-	-	-	
Coumarate	**CMT**	+	+	-	
Beta-Glucoronidase	**BGUR**	-	+	-	-
O/ 129 Resistance (comp.vibrio.)	**O 129R**	+	+	-	+
Glu-Gly-Arg-Arylamidase	**GGAA**	-	-	+	
L-Malate assimilation	**lMLTa**	-	-	-	
Ellman	**ELLM**	-	+	-	
L-Lactate assimilation	**lLATa**	-	-	-	
D-Amygdalin	**AMY**				-
Phosphatidylinositol Phospholipase C	**PIPLC**				-
D-Xylose	**dXYL**				-
Arginine dihydrolase 1	**ADH 1**				+
Cyclodextrin	**CDEX**				-
L-Aspartate Arylamidase	**AspA**				-
Beta Galactopyranosidase	**BGAR**				-
Alpha-Mannosidase	**AMAN**				-
Leucine Arylamidase	**LeuA**				-
Beta Glucuronidase	**BGURr**				-
Alanine Arylamidase	**AlaA**				-
Polymixin B resistance	**POLYB**				+
D-Galactose	**dGAL**				+
D-Ribose	**dRIB**				-
Lactose	**LAC**				-
N-Acetyl-D-Glucosamine	**NAG**				-
Bacitracin Resistance	**BACI**				+
Novobiocin Resistance	**NOVO**				-
Growth in 6.5 % NaCl	**NC6.5**				+
Methyl-B-D-Glucopyranoside	**MBdG**				+
Pullulan	**PUL**				-
D-Raffinose	**dRAF**				-
Salicin	**SAL**				-
Arginine dihydrolase 2	**ADH2s**				-
Optochin Resistance	**OPTO**				+

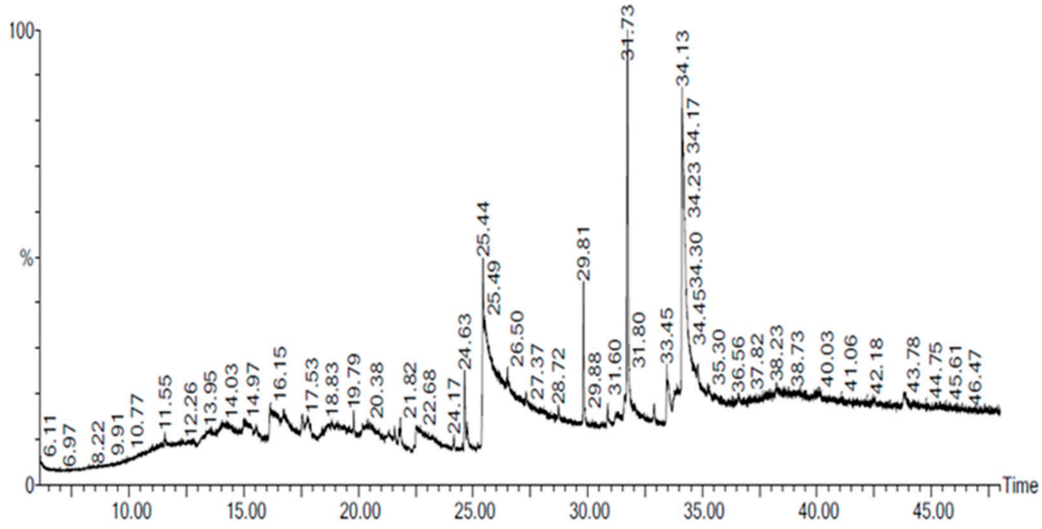

Figure S1. GC–MS chromatograph of ethanol extract of Silybum marianum seeds

Table S4. Active components of *S. marianum* seed ethanol extract identified by means of GC-MS. For each compound, retention time (RT), peak area percentage, molecular formula (MF) and molecular weight (MW).

Peak no.	RT	Area %	Compound name	MF	MW
4	13.95	4.332	d-Mannose	$C_6H_{12}O_6$	180.16
8	14.97	1.876	N-methyl-1-adamantaneacetamide	$C_{13}H_{21}NO$	207.31
12	16.15	14.839	d-Mannitol, 1-decylsulfonyl-(sugar alcohol with sulfur)	$C_{16}H_{34}O_7S$	370.51
14	17.54	0.830	Desulfosinigrin	$C_{10}H_{17}NO_6S$	279.31
21	24.63	0.616	9,12-Octadecadienoic acid, methyl ester, (E,E)-(linolelaidic acid, methyl ester), or (methyl linolelaidate)	$C_{19}H_{34}O_2$	294.47
22	25.44	20.000	9,12-Octadecadienoic acid (Z,Z)-(linoleic acid)	$C_{18}H_{32}O_2$	280.44
27	31.73	3.276	1,2-Benzenedicarboxylic acid, diisooctyl ester (diisooctyl phthalate)	$C_{24}H_{38}O_4$	390.56
28	33.45	3.097	1-Monolinoleoylglycerol trimethylsilyl ether	$C_{27}H_{54}O_4Si_2$	498.89
29	34.13	9.596	Linoleic acid ethyl ester (ethyl linoleate) (mandenol)	$C_{20}H_{36}O_2$	308.50

Table S5. Identified SMILES strings for the nine phytochemical compounds from *Silybum marianum* seed extract: that were detected by GC-MS and retrived form PubChem database of phytochemical constitutions

No.	Compound	SMILES
1	d-Mannose	C(C1C(C(C(C(O1)O)O)O)O)O
2	N-methyl-1-adamantaneacetamide	CNC(=O)CC12CC3CC(C1)CC(C3)C2
3	d-Mannitol, 1-decylsulfonyl- (sugar alcohol with sulfur)	CCCCCCCCCCS(=O)(=O)CC(C(C(C(CO)O)O)O)O
4	Desulfosinigrin	C=CCC(=NO)SC1C(C(C(C(O1)CO)O)O)O
5	9,12-Octadecadienoic acid, methyl ester	CCCCCC=CCC=CCCCCCCCC(=O)OC
6	linoleic acid	CCCCCC=CCC=CCCCCCCCC(=O)O
7	diisooctyl phthalate	CC(C)CCCCCOC(=O)C1=CC=CC=C1C(=O)OCCCCCC(C)C
8	1-Monolinoleoylglycerol trimethylsilyl ether	CCCCCC=CCC=CCCCCCCCC(=O)OCC(CO[Si](C)(C)C)O[Si](C)(C)C
9	mandenol	CCCCCC=CCC=CCCCCCCCC(=O)OCC

Table S6. Physicochemical and ADMET properties of the nine compounds identified in *Silybum marianum* seed extract: (1) d-Mannose, (2) N-methyl-1-adamantaneacetamide, (3) d-Mannitol, 1-decylsulfonyl-(sugar alcohol with sulfur), (4) Desulfosinigrin, (5) 9,12-Octadecadienoic acid, methyl ester, (6) Linoleic acid, (7) Diisooctyl phthalate, (8) 1-Monolinoleoylglycerol trimethylsilyl ether, (9) Mandenol.

#	**1**	**2**	**3**	**4**	**5**	**6**	**7**	**8**	**9**
				**Physicochemical Properties**					
Formula	$C_6H_{12}O_6$	$C_{13}H_{21}NO$	$C_{16}H_{34}O_7S$	$C_{10}H_{17}NO_6S$	$C_{19}H_{34}O_2$	$C_{18}H_{32}O_2$	$C_{24}H_{38}O_4$	$C_{27}H_{54}O_4Si_2$	$C_{20}H_{36}O_2$
MW(g/mol)	180.16	207.31	370.5	279.31	294.47	280.45	390.56	498.89	308.5
#Heavy atoms	12	15	24	18	21	20	28	33	22
Fraction Csp3	1	0.92	1	0.7	0.74	0.72	0.67	0.81	0.75
#Rotatable bonds	1	3	15	5	15	14	16	22	16
#H-bond acceptors	6	1	7	7	2	2	4	4	2
#H-bond donors	5	1	5	5	0	1	0	0	0
LogS (log mol/L)	-0.017	-2.564	-2.116	-0.517	-6.465	-5.23	-7.04	-7.192	-6.596
LogD (log mol/L)	-2.139	2.709	1.038	-0.695	4.646	3.58	5.345	6.399	4.803
LogP (log mol/L)	-2.499	2.301	0.183	-0.76	6.992	6.652	7.494	8.363	7.217
				**Absorption parameters**					
Pgp-inh	0.001	0.035	0.003	0.003	0.001	0	0.968	0.258	0.001
Pgp-sub	0.098	0.001	0.024	0.001	0.028	0.002	0	0.005	0.013
HIA	0.899	0.006	0.938	0.912	0.007	0.01	0.001	0.005	0.003
F(20%)	0.054	0.002	0.996	0.728	0.008	0.009	0.988	0.008	0.008
F(30%)	0.944	0.002	0.972	0.997	0.775	0.549	0.956	0.033	0.729
Caco-2 (log cm/s)	-5.318	-4.62	-5.778	-5.598	-4.551	-4.733	-4.655	-4.811	-4.526
MDCK (log cm/s)	0.001190525	4.71E-05	6.13E-05	0.000211674	1.91E-05	1.92E-05	1.83E-05	2.27E-05	1.86E-05
				**Distribution parameters**					
BBB	0.48	0.982	0.261	0.569	0.245	0.196	0.013	0.001	0.119
PPB %	12.50%	59.27%	74.81%	45.91%	96.84%	98.39%	97.63%	100.90%	97.29%
VDss (L/kg)	0.395	0.879	0.616	0.401	2.926	0.626	1.445	2.796	2.707
Fu %	80.06%	55.91%	27.49%	48.76%	2.08%	1.62%	1.57%	1.01%	2.07%
				**Metabolism parameters**					
CYP1A2-inh	0.01	0.159	0.016	0.02	0.941	0.235	0.134	0.424	0.939
CYP1A2-sub	0.046	0.314	0.092	0.031	0.179	0.171	0.178	0.605	0.166
CYP2C19-inh	0.01	0.815	0.006	0.017	0.569	0.086	0.699	0.435	0.601
CYP2C19-sub	0.15	0.138	0.226	0.059	0.064	0.066	0.06	0.488	0.058
CYP2C9-inh	0.001	0.506	0.001	0.002	0.6	0.43	0.36	0.66	0.637

CYP2C9-sub	0.16	0.613	0.693	0.584	0.947	0.988	0.878	0.905	0.937
CYP2D6-inh	0.002	0.501	0.001	0.001	0.167	0.006	0.116	0.054	0.337
CYP2D6-sub	0.133	0.295	0.028	0.129	0.139	0.086	0.022	0.262	0.098
CYP3A4-inh	0.004	0.435	0.007	0.009	0.692	0.085	0.254	0.678	0.608
CYP3A4-sub	0.01	0.168	0.027	0.019	0.065	0.019	0.088	0.096	0.068
**Excretion parameters**									
CL (ml/min/kg)	1.474	9.954	4.437	1.449	7.742	3.327	9.241	3.424	7.094
T12 (hr)	0.722	0.157	0.476	0.733	0.343	0.628	0.044	0.155	0.265
**Toxicity parameters**									
hERG	0.039	0.007	0.263	0.018	0.1	0.009	0.18	0.158	0.104
H-HT	0.046	0.689	0.023	0.074	0.011	0.013	0.003	0.01	0.004
DILI	0.09	0.027	0.053	0.717	0.022	0.009	0.367	0.014	0.02
Ames	0.203	0.004	0.013	0.336	0.016	0.013	0.002	0.234	0.014
ROA	0.139	0.07	0.007	0.346	0.009	0.01	0.003	0	0.009
FDAMDD	0.002	0.084	0.003	0.005	0.024	0.017	0.004	0.046	0.015
SkinSen	0.04	0.022	0.021	0.042	0.975	0.961	0.933	0.987	0.976
Carcinogenicity	0.013	0.64	0.012	0.572	0.467	0.153	0.334	0.258	0.208
EC	0.003	0.003	0.003	0.003	0.965	0.984	0.02	0.998	0.974
EI	0.025	0.259	0.019	0.012	0.961	0.98	0.98	0.944	0.974
Respiratory	0.03	0.12	0.027	0.245	0.8	0.712	0.052	0.851	0.599
BCF	0.242	1.389	0.368	0.416	2.62	0.898	1.159	3.737	2.841
IGC50 (mM)	1.128	2.187	3.57	1.677	4.997	4.453	5.497	5.5	5.097
LC50 (mM)	0.821	3.685	1.81	2.56	5.339	5.113	5.652	3.821	5.568
LC50DM (mM)	2.23	5.029	2.949	3.99	5.197	4.266	5.159	5.699	5.178
NR-AR	0.496	0.007	0.342	0.053	0.78	0.676	0.032	0.295	0.395
NR-AR-LBD	0.012	0.002	0.002	0.007	0.004	0.005	0.003	0.002	0.005
NR-AhR	0.006	0.014	0.005	0.006	0.007	0.01	0.078	0.004	0.006
NR-Aromatase	0.004	0.005	0.29	0.008	0.147	0.284	0.061	0.88	0.388
NR-ER	0.305	0.077	0.073	0.321	0.097	0.149	0.423	0.031	0.106
NR-ER-LBD	0.083	0.004	0.016	0.011	0.006	0.017	0.273	0.006	0.007
NR-PPAR-gamma	0.003	0.009	0.318	0.004	0.39	0.983	0.005	0.03	0.352
SR-ARE	0.027	0.088	0.202	0.016	0.097	0.321	0.03	0.591	0.136
SR-ATAD5	0.041	0.004	0.006	0.006	0.006	0.005	0.005	0.003	0.003
SR-HSE	0.006	0.436	0.086	0.004	0.683	0.575	0.607	0.803	0.705

SR-MMP	0.006	0.021	0.03	0.011	0.415	0.36	0.119	0.788	0.593
SR-p53	0.004	0.005	0.113	0.003	0.056	0.067	0.006	0.199	0.037
Vol (A^3)	156.517	224.885	365.333	249.934	346.851	329.555	437.082	77585268.41	364.147
Dense (g/cm^3)	1.15	0.921	1.013	1.117	0.848	0.85	0.893	0	0.847
nHA	6	2	7	7	2	2	4	4	2
nHD	5	1	5	5	0	1	0	0	0
TPSA Å^2	110.38	29.1	135.29	122.74	26.3	37.3	52.6	44.76	26.3
nRot	1	3	15	5	15	14	16	22	16
nRing	1	4	0	1	0	0	1	0	0
MaxRing	6	0	0	6	0	0	6	0	0
nHet	6	2	8	8	2	2	4	6	2
fChar	0	0	0	0	0	0	0	0	0
nRig	6	13	2	8	3	3	8	3	3
Flex	0.167	0.231	7.5	0.625	5	4.667	2	7.333	5.333
nStereo	5	0	4	5	0	0	0	1	0
NonGenotoxic_Carcinogenicity	0	0	0	0	0	0	0	0	0
LD50_oral (mg/kg)	0	0	0	0	0	0	0	0	0
Genotoxic_Carcinogenicity_Mutagenicity	0	0	0	2	0	0	0	0	0
SureChEMBL	0	0	0	1	0	0	0	0	0
NonBiodegradable	1	0	0	1	0	0	0	0	0
Skin_Sensitization	1	0	0	0	0	0	0	0	0
Acute_Aquatic_Toxicity	2	0	0	0	0	0	0	0	0
Toxicophores	0	1	1	3	0	0	0	0	0
**Medicinal Chemistry**									
QED	0.29	0.74	0.256	0.145	0.224	0.318	0.271	0.069	0.2
Synth	3.595	3.623	3.665	4.511	2.313	2.292	2.089	3.508	2.305
Fsp3	1	0.923	1	0.7	0.737	0.722	0.667	0.815	0.75
MCE-18	22.667	28.8	7	23.294	0	0	9	8	0
Natural Product-likeness	2.627	-0.44	0.345	1.221	1.03	1.187	0.027	0.784	0.875
Alarm_NMR	0	0	1	2	0	0	0	0	0
BMS	0	0	1	1	0	0	0	1	0
Chelating	0	0	0	0	0	0	0	0	0
PAINS	0	0	0	0	0	0	0	0	0
Lipinski	Accepted	Accepted	Accepted	Accepted	Accepted	Accepted	Accepted	Accepted	Accepted

Lipinski #violations	0	0	0	0	1	1	1	1	1
Pfizer	Accepted	Accepted	Accepted	Accepted	Rejected	Rejected	Rejected	Rejected	Rejected
GSK	Accepted	Accepted	Accepted	Accepted	Rejected	Rejected	Rejected	Rejected	Rejected
GoldenTriangle	Rejected	Accepted	Accepted	Accepted	Accepted	Accepted	Rejected	Rejected	Accepted
Ghose #violations	2	0	0	1	1	1	1	4	1
Veber #violations	0	0	2	1	1	1	1	1	1
Egan #violations	0	0	1	1	1	1	1	1	1
Muegge #violations	2	0	0	0	1	1	2	2	2
Bioavailability Score	0.55	0.55	0.55	0.55	0.55	0.85	0.55	0.55	0.55
Synthetic Accessibility	4.08	3.66	5.37	5.14	3.18	3.1	3.41	5.93	3.34

Abbreviations used in Table S5.

Physicochemical Properties: MW: Molecular Weight, #Heavy atoms: Number of heavy atoms in the molecule, Fraction Csp3: Fraction of sp3-hybridized carbon atoms, #Rotatable bonds: Number of rotatable bonds, #H-bond acceptors: Number of hydrogen bond acceptor atoms, #H-bond donors: Number of hydrogen bond donor atoms, LogD: Distribution coefficient (ratio of concentrations in octanol and water), LogP: Partition coefficient (ratio of concentrations in octanol and water).

Absorption: Measure of the drug's ability to be absorbed into the bloodstream, Pgp-inh: P-glycoprotein inhibitor, Pgp-sub: P-glycoprotein substrate, HIA: Human intestinal absorption, F(20%): Fraction absorbed at 20%, F(30%): Fraction absorbed at 30%, Caco-2: Permeability coefficient in Caco-2 cell monolayers, MDCK: Permeability coefficient in MDCK cell monolayers. **Distribution: Drug distribution in tissues,** BBB: Blood-brain barrier permeability, PPB: Plasma protein binding, VDss: Volume of distribution at steady state, Fu: Fraction unbound in plasma.

Metabolism: Drug metabolism, CYP1A2-inh: Inhibitor of CYP1A2 enzyme, CYP1A2-sub: Substrate of CYP1A2 enzyme, CYP2C19-inh: Inhibitor of CYP2C19 enzyme, CYP2C19-sub: Substrate of CYP2C19 enzyme, CYP2C9-inh: Inhibitor of CYP2C9 enzyme, CYP2C9-sub: Substrate of CYP2C9 enzyme, CYP2D6-inh: Inhibitor of CYP2D6 enzyme, CYP2D6-sub: Substrate of CYP2D6 enzyme, CYP3A4-inh: Inhibitor of CYP3A4 enzyme, CYP3A4-sub: Substrate of CYP3A4 enzyme.

Excretion: Drug excretion, CL: Clearance, T12: Half-life.

Toxicity: hERG: Inhibition of human ether-a-go-go-related gene, H-HT: Human hepatotoxicity, DILI: Drug-induced liver injury, Ames: Ames mutagenicity, ROA: Route of administration, FDAMDD: FDA maximum daily dose, SkinSen: Skin sensitization, Carcinogenicity: Carcinogenic potential, EC: Eye corrosion, EI: Eye irritation, Respiratory: Respiratory toxicity, BCF: Bioconcentration factor, IGC50: Inhibition concentration for 50% of growth, LC50: Lethal concentration for 50% of organisms, LC50DM: Lethal concentration for 50% of fish (Danio rerio) embryos, NR-AR: Nuclear receptor androgen receptor, NR-AR-LBD: Nuclear receptor androgen receptor ligand binding domain, NR-AhR: Nuclear receptor aryl hydrocarbon receptor, NR-Aromatase: Nuclear receptor aromatase, NR-ER: Nuclear receptor estrogen receptor, NR-ER-LBD: Nuclear receptor estrogen receptor ligand binding domain, NR-PPAR-gamma: Nuclear receptor peroxisome proliferator-activated receptor gamma, SR-ARE: Stress-responsive antioxidant response element, SR-ATAD5: Stress-responsive ATAD5, SR-HSE: Stress-responsive heat shock element, SR-MMP: Stress-responsive matrix metalloproteinase, SR-p53: Stress-responsive p53, Vol: Molecular volume, Dense: Molecular density, nHA: Number of hydrogen acceptors, nHD: Number of hydrogen donors, TPSA: Topological polar surface area, nRot: Number of rotatable bonds, nRing: Number of rings, MaxRing: Maximum ring size, nHet: Number of heteroatoms, fChar: Fraction of charged atoms, nRig: Number of rigid bonds, Flex: Flexibility, nStereo: Number of stereocenters, NonGenotoxic_Carcinogenicity: Non-genotoxic carcinogenicity, LD50_oral: Oral median lethal dose, Genotoxic_Carcinogenicity_Mutagenicity: Genotoxic carcinogenicity and mutagenicity, SureChEMBL: SureChEMBL confidence score, NonBiodegradable: Non-biodegradable compound, Skin_Sensitization: Skin sensitization potential, Acute_Aquatic_Toxicity: Acute aquatic toxicity, Toxicophores: Toxicophores present in the molecule.

Medicinal Chemistry: Medicinal chemistry-related parameters, QED: Quantitative Estimate of Drug-likeness, Synth: Synthetic accessibility, Fsp3: Fraction of sp3-hybridized carbon atoms, MCE-18: Medicinal chemistry efficiency-18, Natural Product-likeness: Similarity to natural products, Alarm_NMR: NMR spectrum prediction alarm, BMS: Bristol-Myers Squibb score, Chelating: Chelating potential, PAINS: Pan-Assay Interference Compounds, Lipinski: Lipinski's Rule of Five, Lipinski #violations: Number of Lipinski's Rule of Five violations, Pfizer: Pfizer's Rule of Three, GSK: GlaxoSmithKline's Rule of Three, GoldenTriangle: Golden Triangle rule, Ghose #violations: Number of Ghose's Rule of Five violations, Veber #violations: Number of Veber's Rule of Five violations, Egan #violations: Number of Egan's Rule of Three violations, Muegge #violations: Number of Muegge's Rule of Three violations, Bioavailability Score: Compound bioavailability score, Synthetic Accessibility: Ease of synthesis.
